# Zinner Syndrome—A Rare Cause of Recurrent Epididymitis and Infertility

**DOI:** 10.3390/clinpract11040108

**Published:** 2021-12-09

**Authors:** Magdalena Ostrowska, Magdalena Grześk, Szymon Kaczyński, Dominika Skwara, Kacper Kulik, Filip Kowalski, Adam Ostrowski, Tomasz Drewa

**Affiliations:** Department of Urology, Ludwik Rydygier Collegium Medicum in Bydgoszcz, Nicolaus Copernicus University in Torun, 85-067 Bydgoszcz, Poland; magdalenagrzesk@gmail.com (M.G.); szkaczynski@gmail.com (S.K.); dominikaskwara44@gmail.com (D.S.); kulik.kacper@gmail.com (K.K.); filip.kowalski@cm.umk.pl (F.K.); adam.ostrowski@cm.umk.pl (A.O.); tomaszdrewa@cm.umk.pl (T.D.)

**Keywords:** Zinner syndrome, infertility, kidney agenesis, epididymitis

## Abstract

Zinner syndrome (ZS) is a rare disorder that affects only men. It is characterized by a triad of abnormalities, including unilateral renal agenesis, ipsilateral seminal vesicle cysts, and atresia of the ejaculatory tract. Unfortunately, there is no consensus on the best treatment modality. We describe a case of a young male patient with recurrent epididymitis, dysuria, and frequent urination. In the diagnostic evaluation, we found an extended right seminal vesicle in the ultrasound with hyperechoic fluid inside and an absence of the right kidney. We performed magnetic resonance imaging, computed tomography, and semen analysis confirming Zinner syndrome and deteriorated semen parameters. Urethroscopic evaluation and ultrasound-guided puncture of the seminal vesicle were performed. An abscess was excluded. The cytologic evaluation showed hemosiderophages. Tamsulosin was introduced. We found no signs of relapse in a six-month observation, and the patient had no further symptoms. Therefore, minimally invasive treatment is a feasible option in young patients found with early-stage Zinner syndrome.

## 1. Introduction

Zinner syndrome (ZS) is a rare disorder that affects only men. It is characterized by a triad of abnormalities including unilateral renal agenesis, ipsilateral seminal vesicle cyst, and atresia of the ejaculatory tract. About 200 cases have been described in the literature so far [1]. In this paper, we present a patient with recurrent epididymitis and infertility as the main complaints. Unfortunately, there is no consensus on the best treatment modality. In the discussion, we summarize disease mechanisms, the diagnostic process, and treatment approaches. 

## 2. Case Report

**Figure 1:**
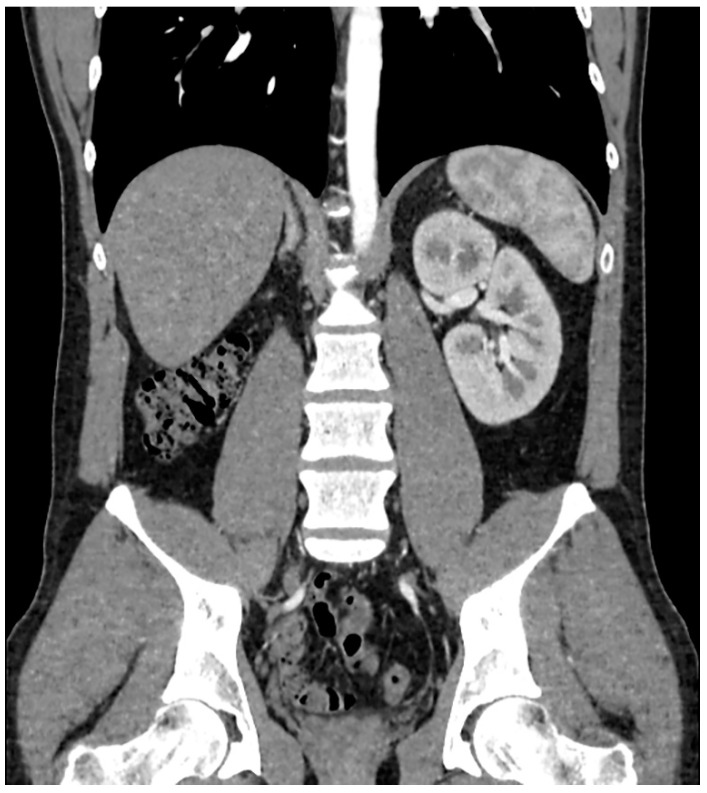
CT scan with contrast showing left kidney and absence of right kidney.

A 31-year-old patient was admitted to the Urology Department to perform an extended diagnostics of seminal vesicle cysts. Since the lesion of the seminal vesicle in ultrasound examination showed an increased density (hyperechoic), it was decided to puncture the vesicle transrectally. In medical history, renal agenesis and recurrent epididymitis were mentioned, which combined were very suggestive of Zinner syndrome. The patient reported additional symptoms such as dysuria and frequent urination. Computed tomography (CT) confirmed agenesis of the right kidney with the enlarged left kidney (Figure 1). 

**Figure 2:**
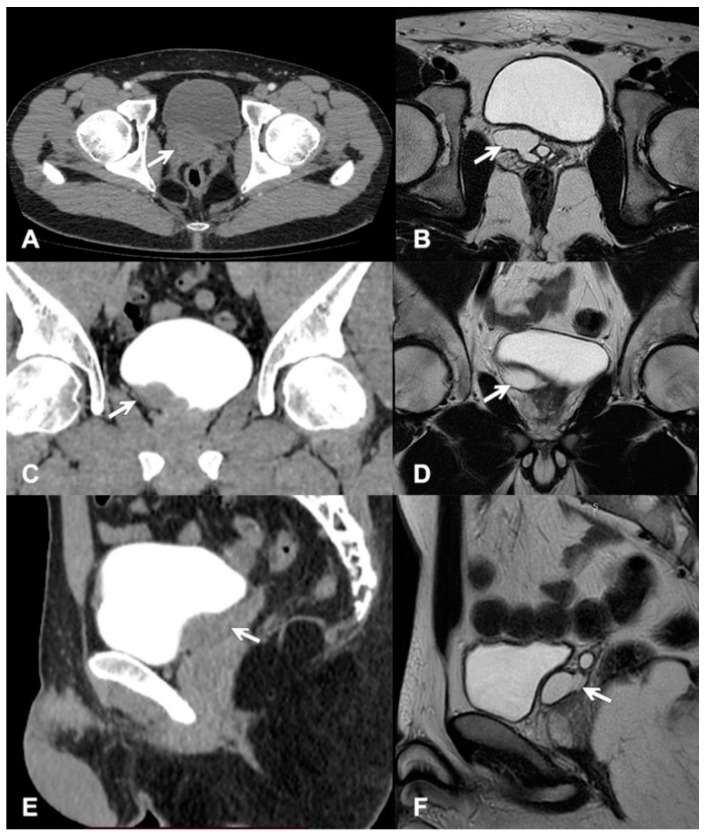
Enlarged seminal vesicle is pointed by the white arrow: (**A**) CT axial plane, (**B**) MRI axial plane, (**C**) CT frontal plane, (**D**) MRI frontal plane, (**E**) CT longitudinal plane, and (**F**) MRI longitudinal plane.

The urinary bladder was modeled from the right posterior side by a 33 × 20 mm cystic lesion in communication with the seminal vesicles (Figure 2A,C,E). For comparison, the magnetic resonance images (MRI) are shown in Figure 2B,D,F. Additionally, the semen examination showed deteriorated parameters (Table 1). 

Urethrocystoscopy was performed under general anesthesia—the urethra, the verumontanum, and the urinary bladder were macroscopically normal. Then, by transrectal ultrasound, we visualized the prostate gland with an enlarged right seminal vesicle. A disposable needle was inserted into the vesicle, and approx. 20 mL of bloody fluid was evacuated. The collected material was sent for cytological examination and culture. The cytology revealed hemosiderophages, and the culture was negative. The patient received an alpha-blocker treatment: Tamsulosin, and was recommended to have regular checkups. During the six-month follow-up, the patient’s quality of life improved—he no longer reported his primary ailments, such as dysuria or epididymitis. However, in the case of persistent complaints, we will consider laparoscopic cyst excision.

## 3. Discussion

Zinner syndrome was first described in 1914. A review of articles from 1999 to 2020 showed that 214 cases are described in the literature [2]. The prevalence rate is estimated at 0.35–0.46/100,000 men [1,2]. Due to the increasing availability of imaging tests, the recognition of ZS is increasing. The characteristic of the syndrome includes unilateral renal agenesis, ipsilateral seminal vesicle cyst, and atresia of the ejaculatory tract [3]. The simultaneous occurrence of anomalies of the kidney and seminal vesicle has been described by embryogenesis. In Zinner syndrome, there is a disturbance in the development of the mesonephric ducts called Wolff’s ducts. These are paired structures differentiating into hemitrigone, bladder neck, urethra, seminal vesicle, vas deferens, ejaculatory ducts, epididymis, paradidymis, and the appendix of the epididymis. The differentiation process takes place between the 4th and 13th week of intrauterine life and is under control by testosterone and the Anti-Mullerian hormone (AMH). The development of the fetal kidney from the mesoderm is dependent on the ureteric bud, which comes from the Wolff’s duct [1,4]. The disturbing process at this stage explains the one-sided and related nature of these changes. The female counterpart of the ZS, which includes Muller ducts, is the Mayer-Rokitansky-Kustner-Hauser syndrome [3]. 

After analyzing the available cases, Liu et al. [5] showed that Zinner syndrome was diagnosed between the neonatal period and the age of 76. In our case, the first symptoms occurred when the patient was 19, but they aggravated at the age of 33. Most often, patients report symptoms between the second and fourth decade of life (along with taking up sexual activity).

When the size of the cyst does not exceed 5 cm, the course is usually asymptomatic. The most common symptoms complicating ZS include frequent urination, dysuria, urgency, and hematuria. Patients also report pain in the perineum, abdomen, and pelvis. The disorders also affect the reproductive system and include, among others, infertility, painful ejaculation, and hematospermia [3,5]. Some publications indicate that up to 45% of patients with Zinner Syndrome will suffer from infertility [6]. The decreased semen parameters may be due to atresia of the ejaculatory tract, the presence of a hematoma in the seminal vesicle, or it may be secondary to inflammation of the genitourinary system [5]. The most common deviations are low semen volume (hypospermia) and no sperm in the ejaculate (azoospermia) [7]. Therefore, according to Kori et al. [4], all patients we intend to introduce surgical treatment should undergo a semen analysis. 

In the diagnostic process of ZS, the first examination is usually ultrasonography. Since the quality and accuracy of ultrasound imaging depend mainly on the examiner’s skill, CT of the abdominal cavity and pelvis seems to be of higher value. CT images will visualize the cysts modeling the bladder and the ipsilateral agenesis of the kidney. The gold standard that most accurately depicts the anatomy of the male reproductive system is MRI [3]. It shows the connection between the cystic lesion and the seminal vesicle, which will confirm the origin of the cyst. In the differential diagnosis, we should include the ejaculatory cyst, ureterocele, and bladder diverticula. MRI is an excellent way to differentiate these changes [3]. Typically, an uncomplicated cyst is anechoic [8]. However, in our case, it was hyperechoic. Zinner syndrome can lead to infectious complications such as an abscess of the seminal vesicle or coexist with other developmental abnormalities or neoplasm [2]. Bearing in mind the possibility of an abscess of the seminal vesicle, we initially decided to aspirate the vesicle’s contents and subject it to cytological analysis and culture. 

It is believed that asymptomatic patients should have only regular checkups. Asymptomatic cysts are usually treated with alpha-blockers. Surgical treatment should be indicated only in patients with symptoms, in case of failure of previous therapy, or when the size of the cyst is greater than 5 cm [6]. To date, there are no official guidelines for the treatment of Zinner syndrome. Due to the limited number of cases, it is currently difficult to state the superiority of one of the methods [4]. Together with the patient, we decided about transrectal cyst aspiration. According to Demaeyer et al., we keep in mind its potential relapse. So this method is suitable but probably shouldn’t be repeated if the desired results are not achieved [9]. The authors agree that only the surgical excision of the cyst and seminal vesicle is 100% effective. Historically, treatment options have included open vesiculectomy via either the transabdominal or transperineal approach. Less invasive procedures such as conventional laparoscopic surgeries are now suggested. The latest articles also describe robotic-assisted surgeries, but the combination of the rarity of the disease and the inaccessibility of advanced tools such as a robot everywhere results in less than 10 cases described in the literature [9]. Unfortunately, there is no consensus on the best treatment modality. As the patient has a single kidney and still risks recurrent urinary tract infection (UTIs), the upper urinary tract has to be closely monitored. Therefore, we planned routine check-ups every six months. In addition, we educated the patient about UTI prevention and the necessity of early treatment in case of recurrence. Each patient should be assessed individually. While some patients need only follow-up, others will benefit from minimally invasive procedures.

## Figures and Tables

**Table 1:** Semen analysis of the patient. Cut of values according to the WHO Laboratory Manual for the Examination and Processing of Human Semen, 5th edition.

Variable	Patient Value	Cut-Off Value
Sperm volume	4.0 mL	>1.5 mL
Sperm concentration	3.8 milion/mL	>15 milion/mL
Total number	15.4 milion/mL	>39 milion/mL
Sperm total movement	5.9%	≥40%
Sperm progressive motility (A + B)	4.7%	>32%
Sperm morphology (correct)	1%	>4%
